# Transcriptomic and Metabolomic Changes Triggered by *Fusarium solani* in Common Bean (*Phaseolus vulgaris* L.)

**DOI:** 10.3390/genes11020177

**Published:** 2020-02-07

**Authors:** Limin Chen, Quancong Wu, Tianjun He, Jianjun Lan, Li Ding, Tingfu Liu, Qianqian Wu, Yiming Pan, Tingting Chen

**Affiliations:** 1 Integrated Plant Protection Center, Lishui Institute of Agricultural and Forestry Sciences, 827 Liyang Stress, Lishui 323000, China; clmit@163.com (L.C.); baiyun12_12@163.com (T.H.); ltf98@126.com (T.L.); pym18867802501@163.com (Y.P.); 2 Plant Protection Station of Songyang County, Lishui 323400, China; syljj888@163.com; 3 Weihai Academy of Agricultural Sciences, No. 411, Tongyi Road, Weihai 311300, China; dyliya1990@163.com; 4 School of Agricultural and Food Science, Zhejiang A&F University, Hangzhou 311300, China; wuqqqqw@163.com; 5 College of Ecology, Lishui University, Lishui, Zhejiang 323000, China; chentingting0626@163.com

**Keywords:** common bean, Fusarium sp, fungus–plant interactions, induced response, transcriptome, metabolome

## Abstract

Common bean (*Phaseolus vulgaris* L.) is a major legume and is frequently attacked by fungal pathogens, including *Fusarium solani* f. sp. *phaseoli* (FSP), which cause Fusarium root rot. FSP substantially reduces common bean yields across the world, including China, but little is known about how common bean plants defend themselves against this fungal pathogen. In the current study, we combined next-generation RNA sequencing and metabolomics techniques to investigate the changes in gene expression and metabolomic processes in common bean infected with FSP. There were 29,722 differentially regulated genes and 300 differentially regulated metabolites between control and infected plants. The combined omics approach revealed that FSP is perceived by PAMP-triggered immunity and effector-triggered immunity. Infected seedlings showed that common bean responded by cell wall modification, ROS generation, and a synergistic hormone-driven defense response. Further analysis showed that FSP induced energy metabolism, nitrogen mobilization, accumulation of sugars, and arginine and proline metabolism. Importantly, metabolic pathways were most significantly enriched, which resulted in increased levels of metabolites that were involved in the plant defense response. A correspondence between the transcript pattern and metabolite profile was observed in the discussed pathways. The combined omics approach enhances our understanding of the less explored pathosystem and will provide clues for the development of common bean cultivars’ resistant to FSP.

## 1. Introduction

Common bean (*Phaseolus vulgaris* L.) is ranked third in China among major food legumes and is mainly distributed in the southwest, northeast, and north of China. Common beans have played an important role in Chinese cropping systems for traditional as well as sustainable agriculture since ancient times [1]. Alone or combined with other stresses, Fusarium root rot caused by *Fusarium solani* f. sp. *phaseoli* (FSP) affects common bean grain yield in major common bean-producing areas in the world [2]. Severe common bean yield losses up to 84% have been reported due to Fusarium root rot (FRR); small-scale farmers in Africa have reported up to 100% crop losses due to FRR [3]. The FRR begins with red-to-brown streaks on taproots, with gradual formation of lesions and necrosis. As the plant develops, the disease becomes more severe and ends in complete rotting of the roots [4]. The best long-term FRR management strategy is to develop resistant cultivars. Hence, it is important to understand the genetic mechanisms controlling FRR resistance in common bean, and how to deploy them to enhance FRR resistance is the current hot topic [4,5]. 

Disease resistance to fungal pathogens is a complex multicomponent system comprising of pathogen detection and signal transduction, followed by a multilayered defense response [6]. Pattern recognition receptors (PRRs) within the cell membrane are the first to respond to invading fungi by detecting pathogen-associated molecular patterns (PAMPs) while wall-associated kinases (WAKs) detect damage-associated patterns (DAMPs). PRRs are a large group of receptors and include NOD-like receptors (NLRs), RIG-I-like receptors (RLRs), Toll-like receptors (TLRs), and C-type lectin receptors (CLRs) [7]. It has been established in many studies that in response to fungal infection, plants have evolved two types of innate immunity responses, i.e., PAMP-triggered immunity (PTI) and effector-triggered immunity (ETI); both are described as a ‘zigzag’ model, with the former having a relatively weak resistance reaction [8]. Post-pathogen detection, a signal transduction system is triggered, involving many pivotal channels, i.e., ion flux, phosphorylation reactions, Ca^++^ concentration fluctuations, and hormone-driven expression of defense responsive genes [9]. With the activation of downstream signaling pathways, plant defense responses, in a multilayered way, are regulated. In these processes, the genes involved in the hypersensitive response (HR), generation of reactive oxygen species, closing of stomata, cell wall modification, and production of defense-related proteins have been reported to be upregulated against fungal pathogens [6].

Several studies on the identification of quantitative trait loci (QTLs) associated with FRR resistance have been conducted and candidate QTLs for marker-assisted selection have been discovered [4,10]. However, the transcriptional response and anatomy of the common bean resistance to FSP have not yet been explored. Host–*F. soalni* pathosystem has only been described in limited crops, i.e., potato, ginseng, soybean, and peas [11,12,13,14]. Host–*F. solani* pathosystem in potato tubers suggested a variety of defense regulation responses, such as the upregulation of genes involved in metabolism, protein fate, cell recue, defense and virulence, interaction with the environment, and cellular communication/signal transduction mechanisms [11]. The study on the *F. solani–Panax notoginseng* pathosystem induced by jasmonate (JA) reported that a large number of genes dedicated to terpenoids biosynthesis, phenylalanine metabolism, and plant–pathogen interaction pathways were upregulated by the exogenous application of methyl-JA. A synergistic action of JA and ethylene (ET) signaling pathways was suggested to positively regulate the defense response in *P. notoginseng* [14]. On the other hand, transcriptomes of *F. solani* have been studied in the *F. solani*–ginseng pathosystem and largely explored pathogen genes related to chitin synthase and cell wall integrity genes [15]. The non-host disease resistance response of pea (*Pisum sativum* L.) to *F. solani* was explored, and pea endocarp tissues were described have a 6-h time window for resistance [12]. While these studies involved transcriptomic approaches to elucidate the possible defense responses in the studied crop plants, the application of the whole-metabolome approach has only been restricted to other fungal–plant pathosystems [16,17].

Combined approaches involving high-throughput transcriptome sequencing and whole-metabolome profiling are improving our understanding in plant–pathogen and plant–insect pest interactions [17,18,19,20]. With the limited amount of information available on the transcriptomic and metabolomic response of common bean to FSP infection, in this study, we employed a combined de novo transcriptome and metabolome analysis to obtain comprehensive insight into the defense responses of common bean to FSP.

## 2. Material and Methods

### 2.1. Plant Growth and in Vivo Inoculations

Common bean seeds of variety Liyun No. 2 were obtained from Lishui Institute of Agricultural Sciences, China. The standard strain of FSP (LS20) was provide by the Lishui Institute of Agricultural and Forestry Sciences. The fungal strain FSP, which belongs to Race 1, was originally isolated from diseased *P. vulgaris* in Longquan, Zhejiang province, China. FSP was incubated on potato-dextrose agar plates at 28 °C for 5–7 days, followed by growth in potato broth on a shaker at 125 rpm at 28 °C for 5–7 days. The suspension concentration was adjusted to 1 × 106 spores/mL with sterile distilled water prior to inoculation. Common bean seedlings were grown in pots filled with sterile vermiculite and clay mixed in a 3:1 vol/vol ratio under normal conditions; 25 °C/18 °C day/night temperatures with a 16-h light/8-h dark photoperiod, and 60% humidity for 5 days. Healthy seedlings at fully expanded trifoliate leaves were then separated into two groups, i.e., CK (control) and FS (FSP treated). The FS groups of seedlings were treated with FSP as reported earlier by [21] while the CK groups of seedlings were supplemented with sterile ultrapure water. Briefly, common bean seedlings (FS group) were inoculated by the root dip method by dipping in a suspension of fungal spores for 15 min, after which the seedlings were returned to the original pots, where they were grown at 28 °C under the same photoperiod. Each group consisted of five seedlings and was monitored for 2, 4, 8, 12, and 18 h. All plants were than evaluated for the disease incidence index and root quantitative characteristics, i.e., root length, fresh weight, dry weight, root volume, dry to fresh ratio, and root density. 

### 2.2. RNA Extraction, Construction of Illumina Library, and Sequencing 

Total RNA was extracted using a Spin Column Plant total RNA Purification Kit following the manufacturer′s protocol (Sangon Biotech, Shanghai, China) [22]. The samples included FS (plants harvested at 18 h post infection) and CK complete roots harvested in triplicate. The purity of the extracted RNAs was assessed on 1% agarose gels as well as by a NanoPhotometer spectrophotometer (IMPLEN, Los Angeles, CA, USA). For RNA quantification, we used a Qubit RNA Assay Kit in Qubit 2.0 Flurometer (Life Technologies, Carlsbad, CA, USA). Further, RNA integrity was assessed by the RNA Nano 6000 Assay Kit of the Agilent Bioanalyzer 2100 system (Agilent Technologies, Santa Clara, CA, USA).

The construction of Illumina sequencing libraries was carried out as previously described (Chen et al., 2019). The cDNA libraries were sequenced on the Illumina HiSeq platform (Illumina Inc., San Diego, USA) by Wuhan MetWare Biotechnology Co., Ltd. (www.metware.cn, Wuhan, China). Raw reads were submitted to the NCBI SRA database under accession numbers PRJNA589754. 

### 2.3. Sequencing Data Analysis 

The clean reads were retrieved after trimming adapter sequences, and removing low-quality (containing > 50% bases with a Phred quality score < 20) reads with unknown nucleotides (more than 1% ambiguous residues N) using the FastQC tool (http://www.bioinformatics.babraham.ac.uk/projects/fastqc/). A GC content distribution check was performed. To stitch clean reads, Trinity was used (Version r20140717, [23]). To avoid contamination of fungal reads, we mapped the reads against the reference genome of *Fusarium solani* [24] and filtered out all positively mapped reads. For hierarchical clustering, Corset was used (https://code.google.com/p/corset-project/). The longest cluster sequence was obtained by clustering with Corset hierarchy as Unigene for subsequent analysis. The assembled unigenes were then aligned with various databases, such as KEGG [25], GO [26], Clusters of Orthologous Groups (COG) [27], PfAM, Swissprot [28], egNOG [29], NR [30], and KOG [31], using BLAST [32] with a threshold of *E*-value < 1.0 × 10^−5^.

The software KOBAS2.0 [33] was employed to obtain the unigene KEGG orthology. The analogs of the unigene amino acid sequences were searched against the Pfam database [34] using the HMMER tool [35] with a threshold of *E*-value < 1.0 × 10^−10^. The sequenced reads were compared with the unigene library using Bowtie [36], and the level of expression was estimated in combination with RSEM [37]. The gene expression level was determined according to the FPKM. 

### 2.4. Differential Expression, Enrichment Analysis, and Real-Time qRT-PCR

The read count was normalized and the EdgeR Bioconductor package [38] was used to determine the differential expression genes (DEGs) between CK and FS, with the fold change > 2 [39] and FDR correction set at *p* < 0.01. GO enrichment analysis was performed using the topGO method based on the wallenius noncentral hypergeometric distribution with *p* < 0.05 [40]. KEGG pathway enrichment analysis of the DEGs was done using KOBAS2.0 [33]. The FDR correction was employed (*p* < 0.05) to reduce false positive prediction of enriched GO terms and KEGG pathways.

Nine DEGs, characterized by interesting expression profiles in response to FSP infection in common bean roots, were randomly selected for qRT-PCR. First strand cDNAs was synthesized from 100 ng of total RNA using the High Capacity cDNA Reverse Transcription Kit (Applied Biosystem, Foster City, CA, USA). Primers were designed using Primer3 Software (http://frodo.wi.mit.edu/primer3/; Table S9) and the specificity was checked by blasting their sequences in the NCBI database. The *Actin* constitutively expressed gene was used as a reference gene [41]. All qRT-PCR reactions were carried out on a Rotor-Gene 6000 machine (Qiagen, Hilden, Germany) with the following thermal cycling profile: 50 °C for 2 min and 95 °C for 2 min, followed by 40 cycles at 95 °C for 3 s and 60 °C for 30 s. Melting curve analysis was performed to verify single product amplification with the temperature ranging from 55 to 95 °C by increasing by 1 °C every step. All reactions were performed in a total volume of 10 μL containing 30 ng of cDNA, 5 μL 1 × SYBR^®^ Select Master Mix (Applied Biosystem), and 0.2 μL (20 μM) of each primer. For each sample, two biological replicates were analyzed in independent runs and a no-template control was included for each gene. Intra-assay variation was evaluated by performing all reactions in triplicate. The quantification cycle (Cq) was automatically determined using Rotor-Gene 6000 Series Software, version 1.7 as reported earlier [42]. 

### 2.5. Metabolome Analysis

#### 2.5.1. Sample Preparation

All procedures related to sample preparation, metabolome profiling, and data analysis were performed at Wuhan MetWare Biotechnology Co., Ltd. (www.metware.cn) following their standard procedures [43]. Briefly, the freeze-dried root samples of FS and CK were crushed to a powder using a MM 400, Retsch grinder. After weighing 100 mg of crushed powder, aliquots were extracted at 4 °C with 0.6 mL of 70% aqueous methanol. To achieve a higher extraction rate, the aliquots were vortexed six times during the extraction process. The aliquots were then centrifuged at 10,000 *g* for 10 min to obtain supernatant after which the samples were filtered using microporous membrane (0.22 μm) and further processed/stored for UPLC-MS/MS analysis.

#### 2.5.2. Chromatographic Mass Spectrometry Acquisition Conditions

The data acquisition instrument system included Ultra Performance Liquid Chromatography (UPLC) (Shim-pack UFLC SHIMADZU CBM30A, https://www.shimadzu.com.cn/, Tokyo, Janpan) and tandem mass spectrometry (MS/MS) (Applied Biosystems 4500 QTRAP, http://www.appliedbiosystems.com.cn/). The liquid phase conditions included column: (1) waters ACQUITY UPLC HSS T3 C18 1.8 μm, 2.1 mm × 100 mm; (2) mobile phase: phase A = ultrapure water (0.04% acetic acid was added), phase B = acetonitrile (0.04% acetic acid was added); (3) elution gradient: 0.00 min B = 5% in comparison, B was linearly increased to 95% in 10.00 min, and maintained at 95% 1 min, 11.00–11.10 min, B was reduced to 5%, and was 5% balanced to 14min; (4) flow rate 0.35 mL/min; column temperature 40 °C; injection volume 4 μL. Whereas the mass spectrometry conditions were as follows: The electrospray ionization (ESI) temperature was 550 °C, the mass spectrometry voltage was 5500 V, the curtain gas (CUR) was 30 psi, and the collision-induced dissociation (CAD) parameter was set high. In the triple quadrupole (QQQ), each ion pair was scanned for detection based on an optimized decolusting potential (DP) and collision energy (CE) [44].

Based on the self-built database MWDB (Metware database) at Wuhan MetWare Biotechnology Co., Ltd. (www.metware.cn), the material was characterized according to the secondary spectrum information. The isotope signal was removed during the analysis, and the repeated signals, including K+ ions, Na+ ions, NH4+ ions, and fragment ions, which are themselves other larger molecular weight substances, were removed.

Metabolite quantification was performed using multiple reaction monitoring (MRM) in triple quadrupole mass spectrometry. In the MRM mode, the fourth-stage rod first screens the precursor ions (parent ions) of the target substance, and excludes the ions corresponding to other molecular weight substances to initially eliminate the interference; the precursor ions break through the collision chamber to induce ionization and break to form a lot of fragment ions, fragment ions. The triple quadrupole filter is then used to select a desired feature fragment ion to eliminate non-target ion interference, which makes the quantification more accurate and repeatable. After obtaining metabolite mass spectrometry data for different samples, peak area integration was performed on the mass spectral peaks of all the substances, and the mass spectral peaks of the same metabolite in different samples were integrated [45].

#### 2.5.3. Metabolomics Data Analysis 

Data matrices with the intensity of metabolite features under FOP and control conditions were uploaded to the Analyst 1.6.1 software (AB SCIEX, Ontario, Canada). For statistical analysis, missing values were assumed to be below the limits of detection, and these values were imputed with a minimum compound value [44]. The relative abundance of each metabolite was log transformed before analysis to meet normality. A Dunnett’s test was used to compare the abundance of each metabolite between control and FOP. The false discovery rate was used for controlling multiple testing. The supervised multivariate method, partial least squares-discriminant analysis (PLS-DA), was used to maximize the metabolome difference between the control and FOP-treated samples. The relative importance of each metabolite to the PLS-DA model was checked using a parameter called the variable importance in projection (VIP). Metabolites with VIP > 1.0 were considered as differential metabolites for group discrimination. Principal component analysis (PCA), hierarchical cluster analysis (HCA), and KEGG pathway analysis were performed in the R software (www.r-project.org).

### 2.6. Co-Joint Analysis of Transcriptome and Metabolome

The differential genes and metabolites were mapped onto the KEGG pathway at the same time. The enrichment results of the differential metabolites and genes were used to show the degree of enrichment pathways [33]. To study the correlation between the genes and metabolites, Corson program in R was used to calculate the Pearson correlation coefficients and were represented as heat map [46].

## 3. Results

### 3.1. Phenotypic Performance of Common Bean Roots under FSP Infection

**Figure 1:**
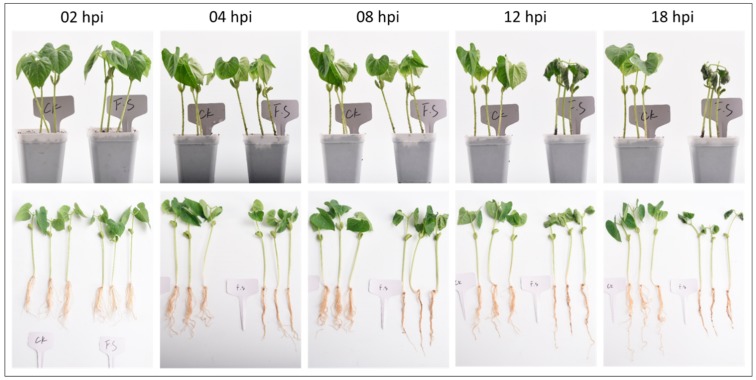
*Fusarium solani* f. sp. *Phaseoli* inoculated (FS) and non-inoculated (CK) common bean seedlings at 2, 4, 8, 12, and 18 h post infection.

The progression of dry root rot in common bean variety Liyun No. 2 infected with FSP was monitored for 2, 4, 8, 12, and 18 h after inoculating the roots. We observed that the disease incidence index reached its maximum (severity rate 5) at 18 h after infection. Three traits, i.e., fresh weight, root volume, and dry to fresh root weight ratio, were also significantly different at 4, 8, and 12 h. The root qualitative traits recorded for each set of seedlings from each time point were compared and it showed that all traits differed significantly in CK (control plants) and FS (infected plants) groups at 18 h (Figure 1; Table S1). These data showed all the treated seedlings at 18 h, confirming the establishment of dry root rot. 

### 3.2. Overview of Transcriptomic Analysis

**Figure 2:**
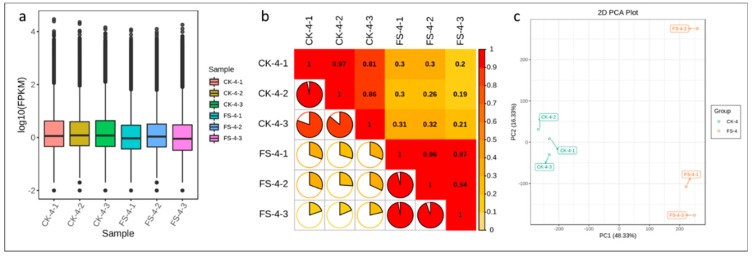
(**a**) Distribution of the gene expression in FSP-infected (FS) and non-infected (CK) common bean roots; (**b**) Pearson correlations between CK and FS replicates; and (**c**) principle component analysis of expressed genes.

The cDNA libraries constructed from CK and FS (plants infected with FSP and harvested at 18 h post infection) common beans were sequenced with the Illumina Hiseq^TM^ high-throughput sequencing platform. After filtering low-quality reads and adapter sequences, a total of 40.19 Gb clean data was obtained consisting of Illumina reads ranging from 41,431,418 to 46,459,376 million/sample (average 44,657,012.33) (Table S2). Functional annotation of all unigenes as annotated to the Kyoto encyclopedia of genes and genomes, non-redundant (NR), Swiss-Port, Trembl, euKaryotic Ortholog Groups (KOG), gene ontology (GO), and pfam databases is presented in Figure S1A; a total of 151,720 unigenes could be annotated. The transcript sequences of common bean roots in our results had 9.76% (*Quercus suber*), 3.24% (*Vigna angularis*), 2.455% (*Vigna radiate* var. radiata), 1.92% (*Cajanus cajan*), 1.65% (*Glycine max*), and 0.91% (*Vigna angularis* var. angularis) similarity with related species (Figure S1B). The clean data of each sample was serialized with the assembled unigene libraries and 87.44–88.17% and 88.37–89.17% reads could be mapped for the CK and FS samples, respectively (Table S3). Overall, the fragments per kilobase of transcript per million fragments mapped (FPKM) for CK was higher as compared to FS, with FPKM > 1 used as a threshold to determine the gene expression (Figure 2a). Pearson correlations between FS-inoculated replicates ranged from 0.2 to 0.32 (Figure 2b). Principal component analysis (PCA) showed that CK and FS samples were separately aggregated, indicating that significant differences existed in the gene expression profiles. The FS-inoculated replicates did not strictly cluster together, suggesting that inoculation within the replicates differed (Figure 2c). 

The GO annotation indicated that 103,528 unigenes were categorized into 59 functional terms in three categories, i.e., biological process (BP), cellular component (CC), and molecular function (MF). Among BP, the cellular amino acid catabolic process, steroid biosynthesis process, and alpha-amino acid catabolic process were the dominant terms. Among MF, amino acid transmembrane transporter activity was the most dominant term followed by oxidoreductase activity acting on the CH-CH groups of donors, NAD or NADP as an acceptor, and sulfur compound binding (Figure S2A). The KEGG functional enrichment showed that in metabolism, metabolic pathways were the most significantly enriched pathways followed by the biosynthesis of secondary metabolites, and biosynthesis of amino acids. In organismal systems, two pathways were significantly enriched, i.e., plant–pathogen interaction and circadian rhythm–plant (Figure S2B).

### 3.3. Differential Gene Expression Analysis

**Figure 3:**
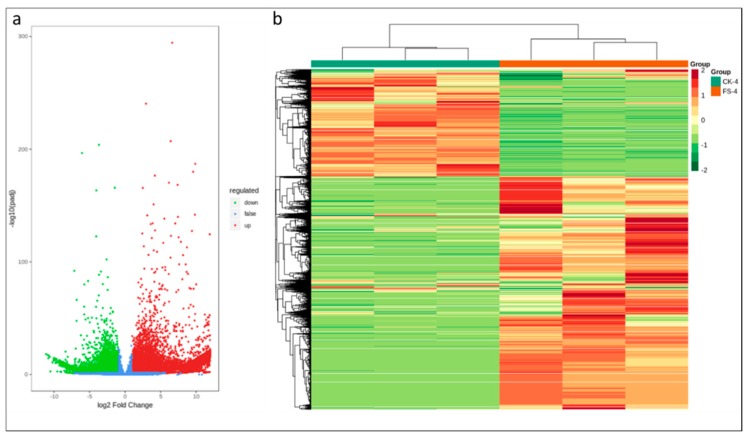
(**a**) Differential gene volcano map and (**b**) differential gene cluster heat map; the abscissa represents the sample name and hierarchical clustering results, and the ordinate represents the differential genes and hierarchical clustering results.

According to DESeq2 analysis |log 2 Fold Change| ≥ 1, and false discovery rate (FDR) < 0.05, a total of 29,722 transcripts were differentially expressed; 7846 were downregulated and 21,876 genes were upregulated (Figure 3a). Of the differentially expressed genes (DEGs), 1511 genes were exclusively expressed in CK while 13,112 DEGs were specific to FS-infected common bean roots. These observations suggest that after the onset of dry root rot (within 18 h of infection), the transcriptional changes in common bean roots becomes intense (Figure 3b).

**Figure 4:**
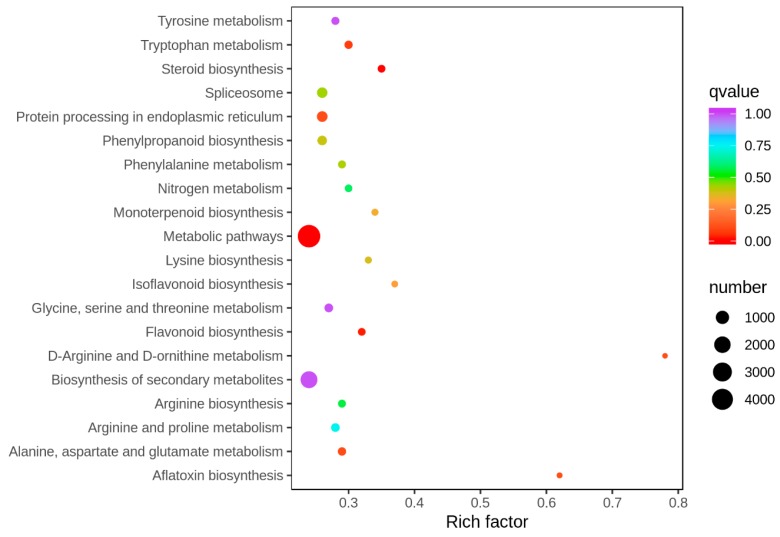
Statistics of KEGG enrichment.

The KEGG analysis revealed the key biological pathways involved in the response to the infection of FSP in common bean roots. Metabolic pathway; biosynthesis of secondary metabolites; ribosome; protein processing in the endoplasmic reticulum; spliceosome; phenylpropanoid biosynthesis; cysteine and methionine metabolism; glycine, serine, and threonine metabolism; glutathione metabolism; and arginine and proline metabolism were the top 10 significantly enriched pathways (Figure 4). 

### 3.4. Transcriptomic Response of FSP-Infected Common Bean Roots

#### 3.4.1. FSP Perception

Chitin is a major constituent of fungal cell walls. Chitin perception is an important feature, hence we observed that two chitin recognition proteins were exclusively and highly up regulated in FS as compared to CK. We observed that 15 other proteins containing GO terms related to cell wall chitin binding, endochitinase/exochitinase activity, and chitin catabolic process were upregulated (Table S4). These proteins were classified (Pfam) as chitin recognition protein (2), glycosyl hdrolases family 18 (GH-18) (8), GDSL-like Lipase/Acylhydrolase family (1), LysM domain (1), transmembrane alpha-helix domain (1), protein kinase domain (1), and phosphotransferase enzyme family (1). Because chitin also binds to homodimers of the Lysine motif (LysM), upregulation of the LysM domain-containing gene might be an FSP perception in our results [47,48]. Chitinases belonging to GH-18 have been reported to function in pathogen recognition and disease resistance in *Zea mays*, *Nicotiana* sp., *Hordeum vulgare*, *Trichoderma atroviride*, *Trichoderma hazianum,* and *Oryza sativa* [49]. Ninety-eight LRR-possessing proteins were upregulated; LRRs bind to extracellular ligands, transmembrane domains necessary for their localization in the plasma membrane, and cytoplasmic kinase domains for signal transduction through phosphorylation [50,51]. Apart from LRRs, PRRs also rely on the regulatory protein brassinosteroid insensitive 1-assoicated receptor kinase 1 (BAK1) as well as somatic embryogenesis receptor-like kinases (SERKs). Hence, we also checked for the expression of these genes and found that 11 BAK1 and two SERKs were upregulated while eight SERKs were downregulated (Table S4).

Other receptors, such as WAKs, are unlike PRRs; WAKs perceive the pathogen as damage to host cell components done by pathogenic enzymes. NLRs, on the other hand, operate in networks and NLRs become activated by direct interaction with pathogen effectors. In our transcriptome, we found that six genes containing cyclic nucleotide-binding domain (3), cobW/HypB/UreG, nucleotide-binding domain (2), and ATP synthase alpha/beta family, nucleotide-binding domain (1) were downregulated. While, 14 genes containing this domain were upregulated, confirming that in FS group, FSP perception is on (Table S4).

#### 3.4.2. Signal Transduction

##### Activation of Signaling Mechanisms

Following fungal chitin and pectin degradation, PRRs, WAKs, and NLRs relay the signal downstream to cascades of signaling pathways and trigger broad-spectrum immunity [17]. These signaling mechanisms are common to many cellular process, i.e., mitogen-activated protein kinases (MAPKs), G-proteins, ubiquitin, and fluctuations in calcium [6]. MAPK cascades are highly conserved signaling modules downstream of receptors/sensors that transduce extracellular stimuli into an intracellular response in plants. We detected that 121 MAPKs were differentially regulated during FSP infection in common bean; 82 of these MAPKs were upregulated. Protein kinases and phosphatases are pivotal in signal transduction processes involved in cell development, differentiation, and most importantly, responses to pathogen attack. In this regard, we found 10 protein kinase inhibitors were differentially regulated (Table S4). Protein phosphorylation has a significant role in signal transduction in disease resistance and the discovery of resistance genes encoding serine/threonine protein kinases confirmed this [52]. We found that a large number of serine/threonine protein kinases were upregulated in FS and some of them were exclusively expressed in FS. Furthermore, we observed that during FSP infection in common bean, several G-proteins and G-protein-coupled receptor (GPCRs) were differentially regulated, confirming functional activation of the G-protein complex. We also observed whether ubiquitination and subsequent protein degradation by proteasomes is functional during FSP infection in common bean. Our transcriptome analysis showed that proteasome-mediated ubiquitin-dependent protein catabolic processes were activated. Finally, to see if receptors triggered fluctuations in calcium ions (Ca^++^) as a signaling response in FSP infection in common bean, we observed calmodulin (CaM), calcium-dependent protein kinases (CKPKs), and calcineurin B-like protein expression. In total, 20 CaMs, 44 CKPKs, and 8 calcineurin B-like proteins were differentially regulated (Table S4). 

##### Systematic Acquired Resistance by Hormones

Downstream of the pathogen detection system, hormone-driven system-acquired resistance adds another layer of defense in plants against pathogen attack. Of the hormones playing a role in downstream signaling pathways, abscisic acid (ABA), auxin, brassinosteroids (BR), cytokinins (CK), ethylene (ET), gibberellin (GA), jasmonic acid (JA), nitric oxide (NO), and salicylic acid (SA) are important ones. In this regard, we found that over 50 DEGs were responsive to FSP infection. We detected that six ET-insensitive genes were downregulated in response to FSP infection. We also detected the levels of genes related to JA signaling, i.e., 22 jasmonate ZIM domain-containing proteins were highly expressed in the FS group of seedlings. Six ethylene-responsive transcription factor 1 genes were upregulated in FS as compared to CK. There were 32 superoxide dismutases, which were differentially expressed with a high fold change value. These expression statistics suggest higher JA levels in FSP-infected common bean roots as compared to CK. Other genes related to JA include lipoxygenases. Thirteen lipoxygenases were differentially regulated; six were upregulated while seven were downregulated, and two allene oxide cyclases were upregulated. Different genes related to auxin, such as auxin-induced proteins, were activated in FS, 15 auxin influx carrier genes were downregulated (three were exclusively expressed in CK), and 9 auxin-responsive GH3 gene family members were differentially expressed (Table S4). 

#### 3.4.3. ROS and Cell Wall Modification of Common Bean Roots After FSP infection

In the first step of invasion, fungi need plant cell wall-degrading enzymes to degrade plant cell walls and facilitate penetration and migration. However, during infection, plant cell wall-related enzymes have also been reported to be active [53]. Among PCWDEs, 2 carbohydrate esterase (*Phavu_009g067900g* and *Phavu_007g146000g*), 13 glycosyl hydrolases (GHs; GH10, GH44, and GH76), and 13 pectate lyases were upregulated, suggesting that FSP infection has triggered these enzymes. An increase in the number and diversity of the enzymes facilitating cell wall breakdown was observed in FS as compared to CK, including GH1, GH28, GH31, GH47, GH88, and the rhamnogalacturonate lyase family. We witnessed that seven cellulose synthase-A genes were downregulated, and five genes were upregulated, suggesting that common bean cell wall is suppressed by the FSP attack and at the same time plant cells respond by upregulating some of the cellulose synthase. Cellulose synthase-like genes were also downregulated in the FS seedling group. Reduced cellulose synthesis is known for invoking a wide range of cellular responses, i.e., activation of lignin synthesis, and JA and ET signaling-mediated defense response [54]. Beta-1,3-glucanases are known for their role in resistance in plants against phytopathogenic fungi [55]. We observed that four beta-1,3-glucanases were upregulated in FS as compared to CK. Two of the four genes were uniquely expressed in FS, indicating that common bean roots respond to FSP by activating these genes. Upregulation of six cellulose synthase-As in FS as compared to CK was also observed. We further observed that six cellulose synthase-like genes annotated as endoglucanase (2), xyloglucan glycosyltransferase 4, cellulose synthase-like protein 92 and beta-mannan synthase were upregulated. Of these, two endoglucanases had 13-fold increased expression in FS as compared to CK (Table S4).

FSP infection triggered the production of peroxidases, which are involved in generation of ROS, which in turn are employed in multiple aspects of resistance [56]. The common bean roots responded to FSP infection by upregulating 19 peroxidases. These genes showed up to 9.96 Log_2_ fold change (*Phavu_008G218600g*) value, indicating that common bean root tissues have activated defense mechanisms against invading FSP. We also observed that four NADPH oxidases were exclusively expressed in FS (8.86- to 14.35-fold). ROS triggers programmed cell death, and H_2_O_2_ moves to nearby cells to initiate the production of compounds to prevent oxidative damage. Eleven primary-amine oxidases were upregulated in FS. These amine oxidases are also involved in ROS production [57].

### 3.5. Validation of DEGs by qRT-PCR 

**Figure 5:**
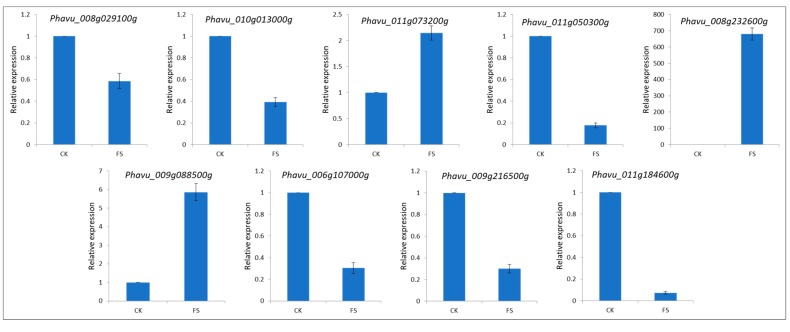
qRT-PCR validation of the selected common bean differentially expressed genes in control (CK) and FS (FSP-infected seedlings) 18 h after infection.

To validate the gene expression data obtained through differential expression analysis, we used qRT-PCR for the expression analysis of nine common bean genes: *Phavu_008g029100g* (BRITTLE CULM1-like 6), *Phavu_010g013000g* (Cytochrome P450 CYP2 subfamily), *Phavu_011g073200g* (Zn-finger transcription factor), *Phavu_011g050300g* (DNA-dependent protein kinase), *Phavu_008g232600g* (Mitogen-activated protein kinase kinase), *Phavu_009g088500g* (Clp amino terminal domain, pathogenicity island component), *Phavu_006g107000g* (domain of unknown function (DUF3535)), *Phavu_009g216500g* (up-frameshift suppressor 2), and *Phavu_011g184600g* (glycosyltransferase family 28 C-terminal domain). The *Actin* gene was used as an internal control to standardize the data, and the amount of target gene transcript-level normalization. All nine selected genes showed similar trend in transcript accumulation, supporting the RNA-seq data (Figure 5). 

### 3.6. Metabolomic Response of FS-Infected Common Bean Roots

**Figure 6:**
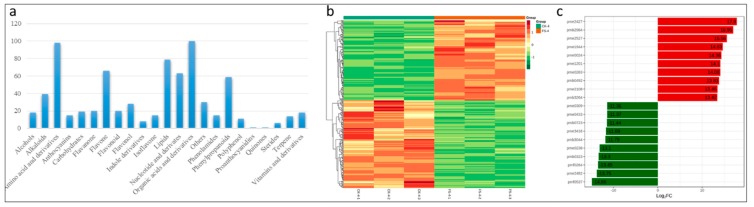
Metabolic profile of FSP-infected common bean roots. (**a**) Major classes of detected metabolites in common bean roots; (**b**) a heatmap hierarchical clustering of the detected metabolites in FS and CK groups; and (**c**) the top 10 differentially expressed metabolites.

In the present study, common bean roots infected with FSP along with CK were used for metabolic profiling as described earlier [17,58]. In total, 743 metabolites were successfully detected in common bean roots (FS and CK) (Table S5). These detected metabolites could be grouped into 22 major classes (Figure 6a). Partial Least Squares Discriminant Analysis (PLS-DA) was applied to evaluate the differences in metabolites between FS and CK. The established PLS-DA model showed food fitness (R2X = 0.661, R2Y = 0.999) and predictability (Q^2^ = 0.977) (Figure S3A). Of these 743 detected metabolites, 300 were differentially expressed in FS vs. CK (Table S6); 143 were upregulated and 157 downregulated. Based on the quantitative metabolite profiles of FS vs. CK, heatmap hierarchical clustering could be clearly distinguished (Figure 6B).

The top 10 most differentially expressed metabolites included N-Acetyl-L-tyrosin, Acetyl-eriodictyol O-hexoside, L(+)-Ornithine, D-Mannitol, 2-Aminoethanesulfonic acid, Phloretin, 5-Methyluridine, “N’,N’’’’,N’’”-p-coumaroyl-cinnamoyl-caffeoyl spermidine”, L-Carnitine, and Glutathione oxidized. In another study on *F. oxysporum* early infection on common bean roots, both N-Acetyl-L-tyrosine and D-Mannitol were also among top 10 upregulated metabolites [17]. 

We further functionally annotated the differentially accumulated metabolites using the KEGG database. The most significantly enriched pathways were metabolic pathways, biosynthesis of secondary metabolites, ABC transporters, biosynthesis of amino acids, flavonoid biosynthesis, isoflavonoid biosynthesis, purine metabolism, and arginine and proline metabolism (Table S7).

**Figure 7:**
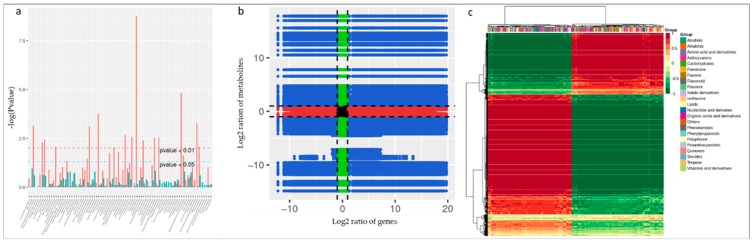
(**a**) Joint KEGG enrichment *p*-value histogram, (**b**) quadrant diagram representing the association of transcriptomic and metabolomic variation in FS vs. CK; the black dotted lines represent the differential thresholds. Outside the threshold lines, there were significant differences in the gene/metabolites, and within the threshold lines are shown the unchanged gene/metabolites. Each point represents a gene/metabolite. Black dots = unchanged genes/metabolites, green dots = differentially accumulated metabolites with unchanged genes, red dots = differentially expressed genes with unchanged metabolites, and blue dots = both differentially expressed genes and differentially accumulated metabolites. (**c**) Correlation coefficient cluster heat map (>0.8).

The co-joint KEGG enrichment analysis showed 82 co-mapped pathways (Table S8). Interestingly, of these co-mapped pathways, tyrosine metabolism, arginine biosynthesis, phenylalanine metabolism, stilbenoid, diarylheptanoid and gingerol biosynthesis, arginine and proline metabolism, and alanine, aspartate, and glutamate metabolism were the most significantly enriched pathways (Figure 7a). The Pearson correlation coefficients for nine quadrants are shown in Figure 7b. The third and the seventh quadrants show that the gene and metabolite differential expression patterns are consistent; genes are positively related to the regulation of metabolite, and/or changes in metabolites may be positively regulated by the genes. DEGs and differentially accumulated metabolites (DAMs) having Pearson correlation coefficients (PCC) higher than 0.8 were further selected and are represented as a heat map (Figure 7c). 

## 4. Discussion

There is a growing interest in linking transcriptomics with other omics tools, including metabolomics, proteomics, and microbiomics. We used an integrated transcriptomics and metabolomics approach to understand the perception, signaling, and defense response in an FSP–common bean pathosystem. These results increase our understanding of the mechanisms underlying the early response of common bean to FSP infection. 

### 4.1. FSP is Perceived by PRRs, WAKs, and NLRs, Which Activate Multiple Signaling Mechanisms 

A suit of cellular receptors, including PRRs, WAKs, and NLRs, detect PAMPs, DAMPs, and effectors that pathogens use to facilitate infection, respectively [6]. Chitin is an essential component of the carbohydrate skeleton of the FSP cell wall. FSP chitin xylanase is identified by plant resistance-like gene family members (chitin oligosaccharide elicitor-binding protein (CEBiP)) and through plasma membrane receptors [59]. The 17.29- and 13.16-fold upregulation of two chitin recognition proteins (Cluster-30876.53339 and Cluster-5217.0, respectively) and a CEBiP (CEBip-LYM2; Cluster-30876.43128) suggested that FSP was perceived by common bean roots at the studied time point. Furthermore, it is also known that in *Arabidopsis*, chitin binds to homodimers of the Lysine motif (LysM) to induce typical immune responses, and also triggers plasmodesmata closure [47,48]. Thus, it is possible that common bean may also employ the same mechanism for the perception of FSP. In order to bind with FSP ligands, common bean must have LRR possessing PRRs. The upregulation of a large number of LRRs in FSP-infected common bean roots at the studied time point confirms that these proteins are active and possibly bind to FSP elicitors. These findings need further detailed interaction of LRRs and FS elicitors [50]. Our results showing the upregulation of BAK1, SERKs, and NRLs are in a strong agreement with previous studies and confirm that common bean utilizes its cellular receptors for the early perception/detection of invading FSP [60,61,62].

The initial touching and perception by plant receptors rapidly triggers the signal transduction process involving multi-level gene expression patterns [63]. The differential regulation of MAPKs at the studied time point in common bean roots suggests that FSP (extracellular stimuli) produces an intracellular response in infected tissues. The downregulation of some of the MAPKs recorded in our transcriptome results might be due to the fact that the defense response can also be downregulated by MAPK signaling; pathogens have evolved effectors that interfere with MAPK signaling and suppress resistance [64]. Fungal elicitors trigger rapid and transient protein phosphorylation in plants [52]. So, an immediate response is the inhibition of early biochemical responses (produced by elicitors) by protein kinase inhibitors. The contrasting expression of protein kinase inhibitors and the upregulation of serine/threonine protein kinases in FS as compared to CK observed in our transcriptome confirmed common bean roots actively transduce signals and activate defense-related proteins [65]. It is known that protein phosphorylation is associated with the presence of Ca^++^ involved in the signal transduction processes. So, the activation of CaM, CKPKs, and calcineurin B-like proteins at 18 h after FSP infection confirmed the processing of downstream signals to activate the MAPKs cascade’s initiation and ROS production [66]. Apart from MAPK, protein phosphorylation, and Ca^++^ associated signaling, the heterotrimeric G-protein and G-protein-coupled receptor (GPCR) system processes signals during pathogen attack [67]. The fact that two GPCRs (Cluster-30876.45183 and Cluster-30876.45184) were exclusively expressed in FS confirms that G-protein heterotrimer is activated in common bean roots infected with FS. Once G-proteins are activated, they transduce extracellular signal from GPCRs. Our results are strongly in agreement with this, as we observed upregulation of 11 G-protein alpha subunits, and exclusive expression of eight G-protein beta subunits (Table S4) [68].

The role of plant hormones in plant defense against *Fusarium* species is obvious [17,69,70]. A previous report demonstrated the upregulation of JA biosynthesis-related genes in an *F. solani*–*P.notogensing* pathosystem [14]. Similar to these results, we observed upregulation of eight hydroxymethylglutaryl-CoA synthases. Three of these (Cluster-30876.52083, Cluster-30876.47713, and Cluster-30876.52082) were exclusively expressed in FS. These results clearly indicate that the JA pathway transduces exclusive signals in the FSP–common bean pathosystem. Similarly, genes related to ET and auxin were differentially regulated in FS vs. CK. The emerged synergistic response of hormones in signal transduction during FSP infection opens up new avenues for understanding the crosstalk between FRR resistance and hormonal signaling. 

### 4.2. FSP Infection Triggered ROS Production in Common Bean Roots

A hypersensitive response leads to a planned cell death in the surrounding area of pathogen infection. On the other hand, fungal infection triggers the production of several enzymes, including peroxidases, in order to generate ROS [71]. Twenty-six peroxidases were upregulated in FS. Of these 26 peroxidases, 7 were exclusively expressed in FS (Cluster-30876.50326, Cluster-30876.45040, Cluster-30876.39585, Cluster-30876.28543, Cluster-19331.0, Cluster-30876.50445, and Cluster-33418.0). These results clearly indicate that FSP infection triggered ROS production in common bean. NADPH oxidases are required for the production of superoxide, which is used to generate hydrogen peroxide (H_2_O_2_). Studies have shown that NADPH oxidases are regulated by PRR-associated kinases [72]. The fact that NADPH oxidases were exclusively expressed in FS indicates the regulation of NADPH oxidases during FSP infection in common bean. We also observed that primary amine oxidases were upregulated in FS, suggesting the production of ROS, programmed cell death, and production of compounds to prevent oxidative damage [57]. 

### 4.3. Cell Wall Acts as a Barrier Against FSP Infection in Common Bean

Similar to other crop plants, it is also known that common bean uses the cell wall as a barrier against pathogenic attack while it is known that fungal pathogens employ plant cell wall-degrading enzymes to destabilize and degrade the host cell wall. We observed whether within the host, cell wall-degrading enzymes are expressed during FSP infection or not. In this regard, the higher expression of GH10, GH44, and GH76, and increased diversity of enzymes belonging to gene families GH1, GH28, GH31, GH47, and GH88 clearly indicate that FSP has successfully established itself in common bean and a weakening response has started [17]. As most plant cell walls are based on a network of cellulose microfibrils and hemicelluloses [73], the upregulation of cellulose synthase-As, cellulose synthase-like enzymes, and other cell wall-strengthening enzymes in FS indicates that common bean cells reinforce cell wall polymers [74]. Callose, like cellulose, is another cell wall polymer that increases its strength, particularly during disease incidence. In this regard, five callose synthases were upregulated in FS. This upregulation could be a defense response in common bean similar to *Arabidopsis*, where the accumulation of callose deposits at the sites of fungal penetration were reported, hence callose synthesis during FSP attack makes a positive contribution to immunity [75,76].

### 4.4. FSP Infection Triggered Different Metabolic Responses in Common Bean

The production of specific metabolites in response to pathogen infestation can elevate the plant’s ability to overcome biotic stress [77]. Our metabolomics results have highlighted differential changes in 23 major classes of metabolites. The differential alteration in primary metabolites, such as amino acids and sugars, suggests that energy metabolism is being manipulated. Energy is critical during defense response execution in plants because of the expression of hundreds and thousands of genes are active in multiple defense pathways [78]. It is also known that sugars affect disease susceptibility and at the same time play a pivotal role in innate defense pathways [79]. An increase in the amount of carbohydrates (D-(+)-sucrose, galactinol, and melibiose) in FS suggests a regulatory role of these sugars in metabolic reprogramming during the early onset of FRR in common bean (Table S6) and that carbohydrate metabolism has a role in the regulation of the defense response [80]. Supportive evidence could be derived from an earlier report on an *F. oxysporum*–chickpea pathosystem [81]. Nitrogen plays an essential role during pathogen–plant interaction. It is known that once a pathogen infects the plant, nitrogen is mobilized, which leads to modulation of the amino acid concentration [82,83]. This is also relevant to our results in the FSP–common bean pathosystem as we observed the upregulation of amino acids and derivatives. Many defense-related compounds are derived from amino acid and their derivatives. The 31 upregulated amino acids and derivatives support the results of the KEGG enrichment analysis that biosynthesis of secondary metabolites was a highly enriched pathway after metabolic pathways (Tables S6 and S7). The results showing the upregulation of N-acetlyl-L-tyrosin, L-cystathionin, glutathione oxidized, glutathione reduced form, 5-aminovaleric acid, and Nα-Acetyl-L-arginine are in agreement with our previous work on the *F. oxysporum*–common bean pathosystem [17]. This suggests that common bean responds to fungal pathogens in a similar manner and these compounds play significant roles in defense against *Fusarium* species. 

Plants respond to pathogens by activating several mechanisms leading to the production of flavonoids [81]. The common bean metabolomic response to FSP infection also showed that two of the significantly enriched pathways were flavonoid biosynthesis and isoflavonoids biosynthesis (Table S6). Proline metabolism plays an important role in redox buffering, energy transfer, and resistance against pathogens in plants [84]. Our metabolome KEGG enrichment chart showed that arginine and proline metabolism was among the top 10 significantly enriched pathways (Table S7). Previously, it has been shown that *Arabidopsis* exhibited a hypersensitive response against *P. syringae* infection, leading to increased proline [85]. The upregulation of the key genes involved in proline metabolism, i.e., pyrroline-5-carboxylate synthetase (Cluster-30876.30681) and pyrroline-5-carboxylate reductase (Cluster-30876.60313), suggests that common bean also accumulates higher proline levels in response to FSP infection (Table S4). 

## 5. Conclusions

Our combined approach of the transcriptomic and metabolomic response of common bean to FSP infection suggested that metabolic pathways are the most significantly enriched pathways. Common bean adopts a zigzag model as a defense response against FSP. The fungal pathogen is perceived by receptors followed by the activation of signaling pathways and a multilayered defense response, i.e., MAPK signaling, protein phosphorylation, G-protein complex activation, and fluctuations in Ca^++^ ions. The defense responses included cell wall modification, ROS production, and hormone-driven system-acquired resistance. FSP triggered different metabolic pathways, including energy metabolism, nitrogen mobilization, increase in sugars, biosynthesis of secondary metabolites, and arginine and proline metabolism. This study gives number of candidate genes and metabolites for further characterization. Further investigations on the detailed functional characterization of these genes will be helpful in breeding programs focusing on disease resistance in common bean.

## Supplementary Materials

The following are available online at https://www.mdpi.com/2073-4425/11/2/177/s1, Figure S1: Unigene annotation chart and (B) NR annotated pie chart showing similarity between the transcript sequencings of *Phaseolus vulgaris* L. with other species, Figure S2: (A) GO enrichment analysis of differentially expressed genes (top 50) and (B) KEGG annotation of differentially expressed genes, Figure S3: PLS-DA model, Table S1: Comparison of means for root quantitative data, Table S2: Summary of sequencing output statistics, Table S3: Mapping results of RNA-Seq data from inoculated and non-inoculated common bean seedlings, Table S4: Complete list of differentially expressed genes regulated by infection of FS at 18 h after infection in comparison with control, Table S5: List of all detected metabolites, Table S6: List of all differentially expressed metabolites, Table S7: KEGG annotation of differentially expressed metabolites, Table S8: Co-mapped KEGG pathways between transcriptome and metabolome data from FS vs CK, Table S9: List of primers used for qRT-PCR.
